# Supplementary material for: Hierarchical graph learning for protein–protein interaction
Source: Nat Commun. 2023 Feb 25;14:1093. doi: 10.1038/s41467-023-36736-1 (PMC9968329; doi:10.1038/s41467-023-36736-1)
Supplement: Supplementary file 1 — Supplementary Information [file 41467_2023_36736_MOESM1_ESM.pdf]

Supplementary Information

Hierarchical Graph Learning for Protein-Protein Interaction

Ziqi Gao, Chenran Jiang, Jiawen Zhang, Xiaosen Jiang, Lanqing Li,

Peilin Zhao, Huanming Yang, Yong Huang and Jia Li

Correspondence to: yonghuang@ust.hk; jialee@ust.hk.

Contents

Supplementary Figure 1. Model architectures and training curves……………………….2

Supplementary Figure 2. Performance of various GNN paradigms……………………3

Supplementary Figure 3. Model effectiveness and efficiency…………………………4

Supplementary Figure 4. Model robustness against protein structure errors…………...5

Supplementary Figure 5. Model performance on various PPI types……………………6

Supplementary Figure 6. Interpretability of identifying binding surfaces……………...7

Supplementary Figure 7. Interpretability of identifying catalytic sites………………...8

Supplementary Figure 8. Empirical study on the cutoff distance……..………………...8

Supplementary Figure 9. Model ability to handle OOD data…...…...………………...14

Supplementary Table 1. Unreliability statistics of created data.........…………………....9

Supplementary Table 2. False discover on reliable data………....…...……………......9

Supplementary Table 3. False discover on unreliable data…...………..……………....9

Supplementary Table 4. Easily available feature options……...…...………………...10

Supplementary Table 5. Feature selection results…………………...………………...11

Supplementary Table 6. Generalization results on 3 distinct classes…...………...…...13

Supplementary Table 7. Robustness results on 3 distinct classes…...………………...14

Supplementary Method 1. The process for feature selection………...………………...10

Supplementary Method 2. In-depth discussion on protein domain data...…...………..12

Supplementary Method 3. Additional experiments on OOD datasets......…...………..13

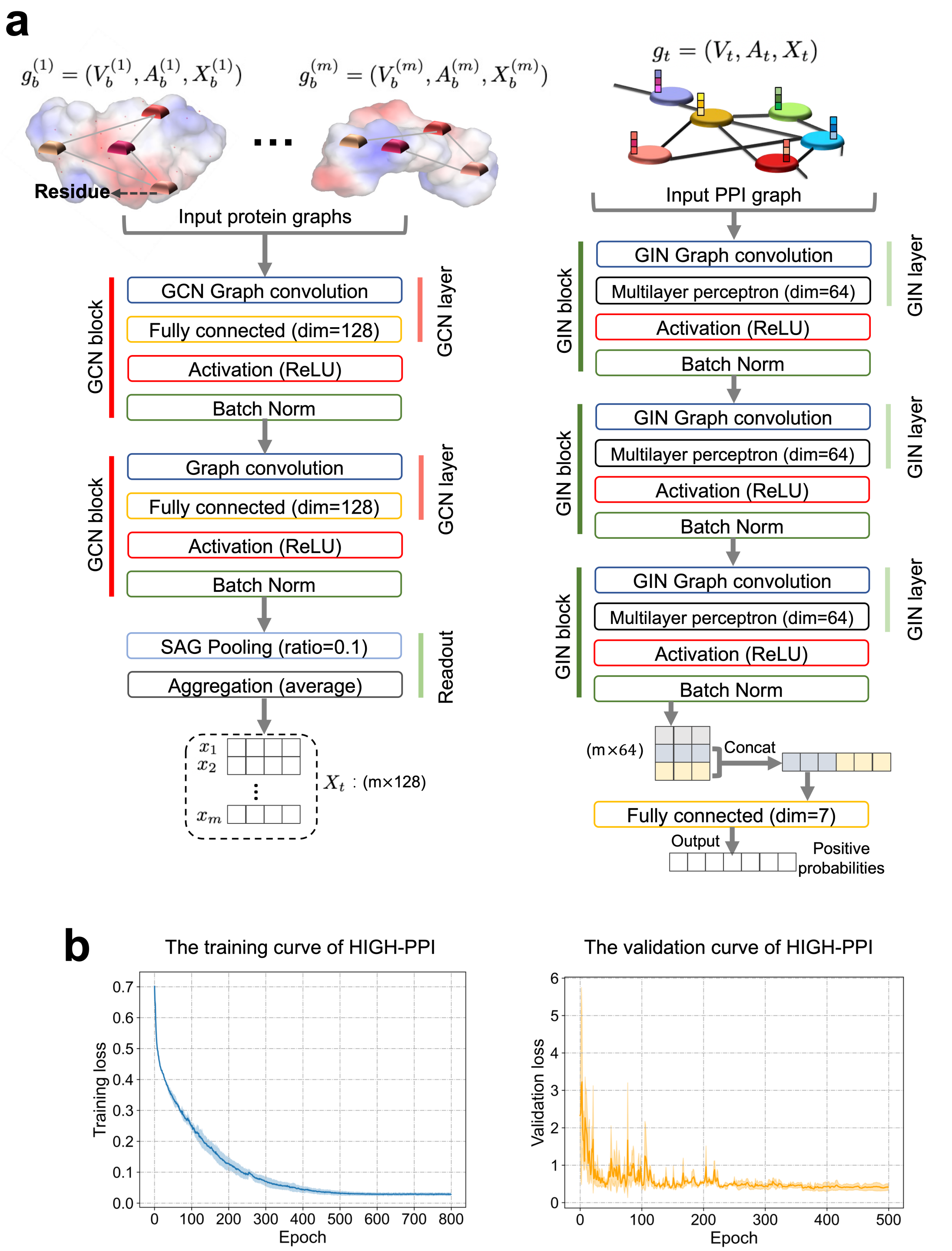

Supplementary Fig. 1. Architecture of the proposed HIGH-PPI model. **(a)** Left: BGNN in HIGH-PPI to obtain protein graph embeddings. Taking m protein graphs as inputs, BGNN computes an embedding matrix $X_t \in \mathbb{R}^{m\times 128}$. Right: TGNN in HIGH-PPI to obtain final PPI positive probabilities by taking the PPI network topology and output X_t of BGNN as inputs. We illustrate the output dimensions of all learnable layers. **(b)** The training (left) and validation (right) curve on the STRING dataset with HIGH-PPI. The curves are depicted on five runs. Error bands represent standard deviation of the mean.

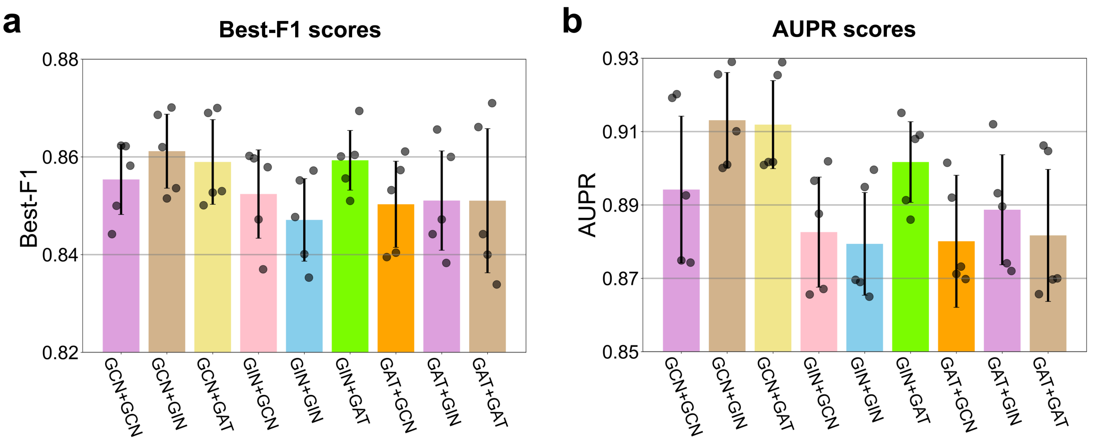

Supplementary Fig. 2. Performance of HIGH-PPI with three different GNN layers. Results of mean and standard deviations in terms of best-F1 **(a)** and AUPR **(b)** under five independent runs. We consider three commonly used GNN layers (GCN, GIN and GAT) for BGNN and TGNN respectively. For example, 'GCN+GAT' represents employing GCN for BGNN and GAT for TGNN. Error bars represent standard deviation of the mean. Source data are provided as a Source Data file.

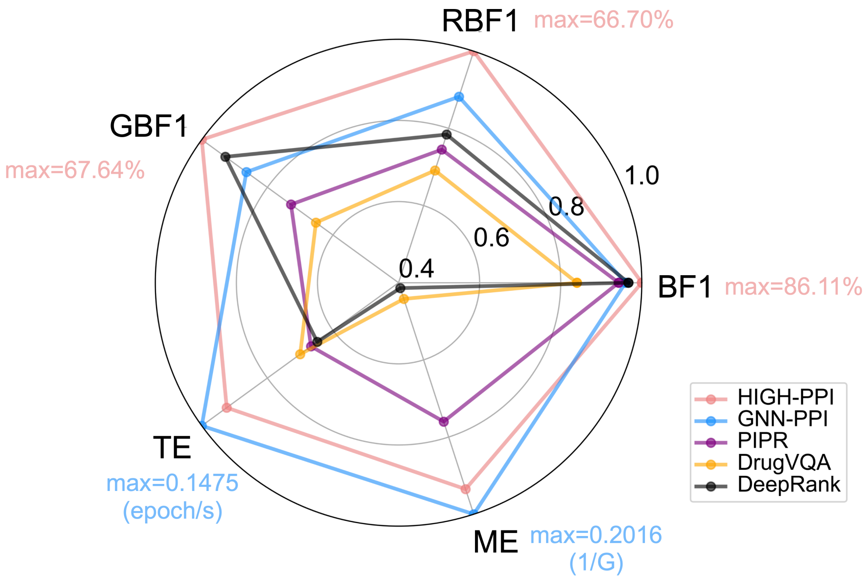

Supplementary Fig. 3. Comparison over five metrics among five baseline methods, GNN-PPI, PIPR, DrugVQA, DeepRank and our proposed HIGH-PPI on SHS27k dataset. Five metrics at the vertices of the radar chart include: best-F1 score (BF1), robustness best-F1 score (RBF1), generalization best-F1 score (GBF1), time efficiency (TE) and memory efficiency (ME). Note that we obtain RBF1 scores by testing the best-F1 of all baselines under a perturbation ratio of 0.4 (see Fig. 2b), and GBF1 scores by testing the best-F1 of all baselines under the 'BFS-0.3' OOD case (see Fig. 2c). For ease of visualization, we normalize each performance score by dividing by the maximum score across methods at each metric. Source data are provided as a Source Data file.

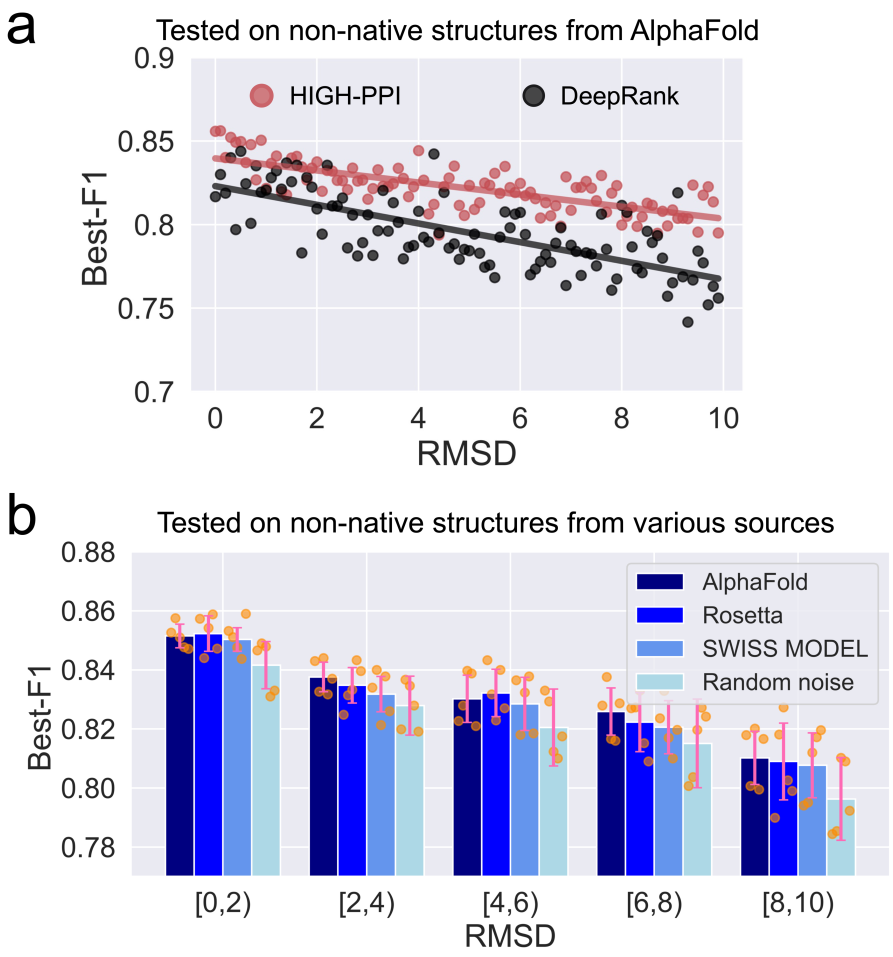

Supplementary Fig. 4. (a) Comparison of the robustness of HIGH-PPI (hierarchical graph) and DeepRank (3D CNN) against protein structure errors. Both models are trained on native structures and tested on structures of different average RMSD. We retrieve all structure information from AlphaFold. **(b)** The impact of protein structure data from various sources on the performance of HIGH-PPI. Our model is trained on native protein structures and tested on structures by using AlphaFold, Rosetta, SWISS MODEL and adding noise to the native ones (Random noise). Bar charts show the best-F1 scores averaged over 5 independent runs and error bars represent standard deviation results. Source data are provided as a Source Data file.

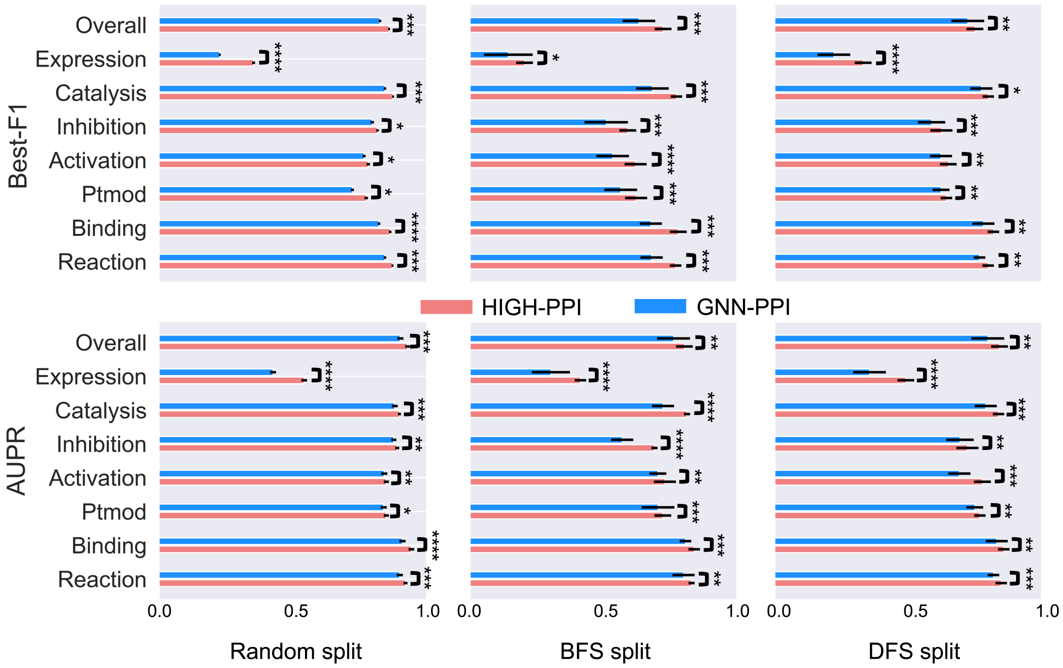

Supplementary Fig. 5. Experiments on native protein structures under three dataset split scenarios show a difference in performance of our method vs. the second-best one (GNN-PPI). We consider a random split and two out-of-distribution (OOD) split manners (BFS and DFS). For each PPI type, we show the performance measured by the best-F1 and AUPR scores averaged over 5 independent runs. Error bars represents standard deviation results. We adopt two-sided t-test for significance analysis. Source data are provided as a Source Data file.

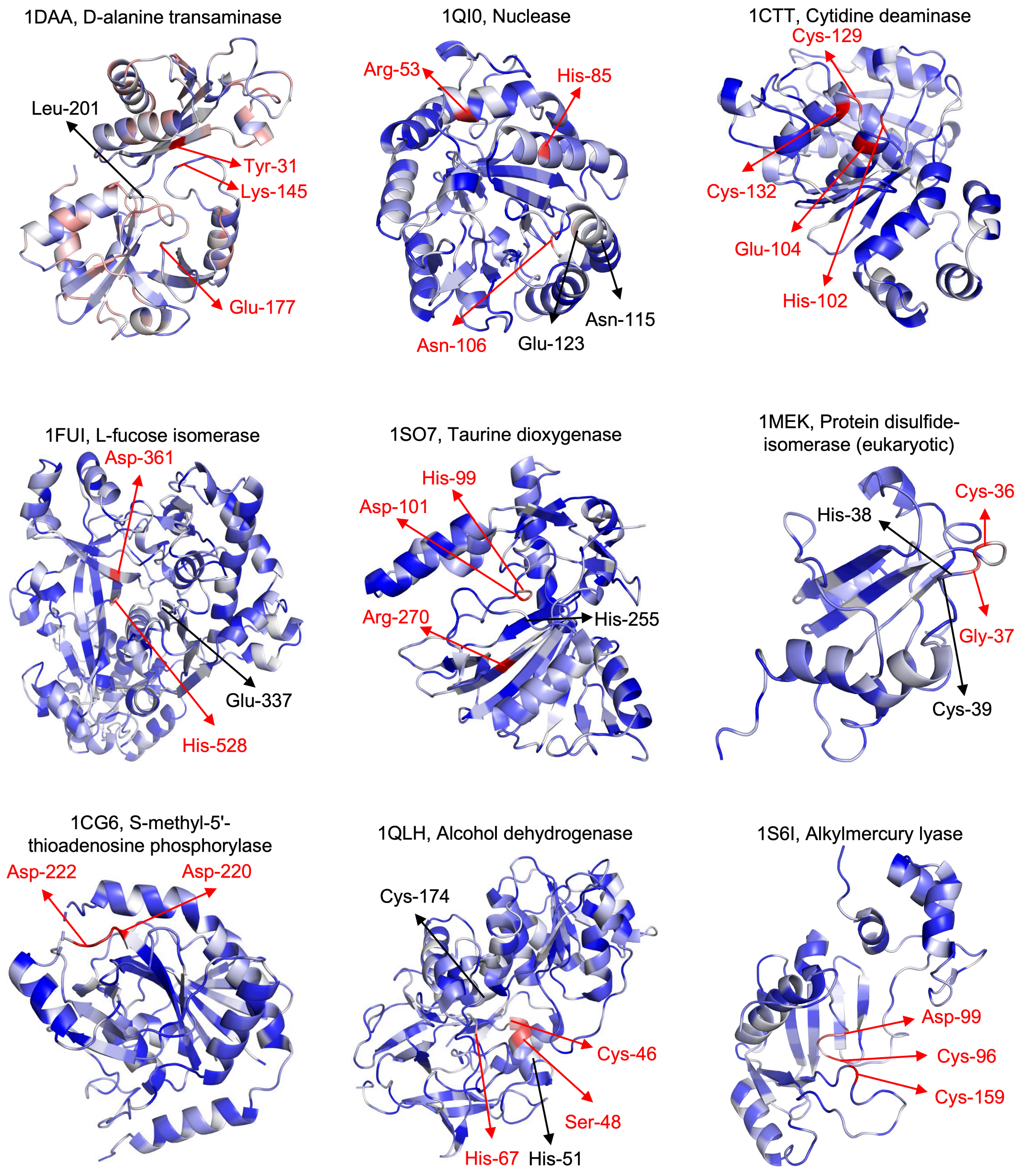

Supplementary Fig. 6. Interpretability of HIGH-PPI for identifying catalytic sites mapped onto the 'cartoon' models of tested proteins. We focus on PPI pairs of the 'catalysis' type and define the enzymes as the query protein. The ground truth of catalytic labeled on each cartoon representation is retrieved from the CSA database. We highlight the sites that are correctly identified in red (25 sites in total) and that are not identified in black (9 sites in total). Source data are provided as a Source Data file.

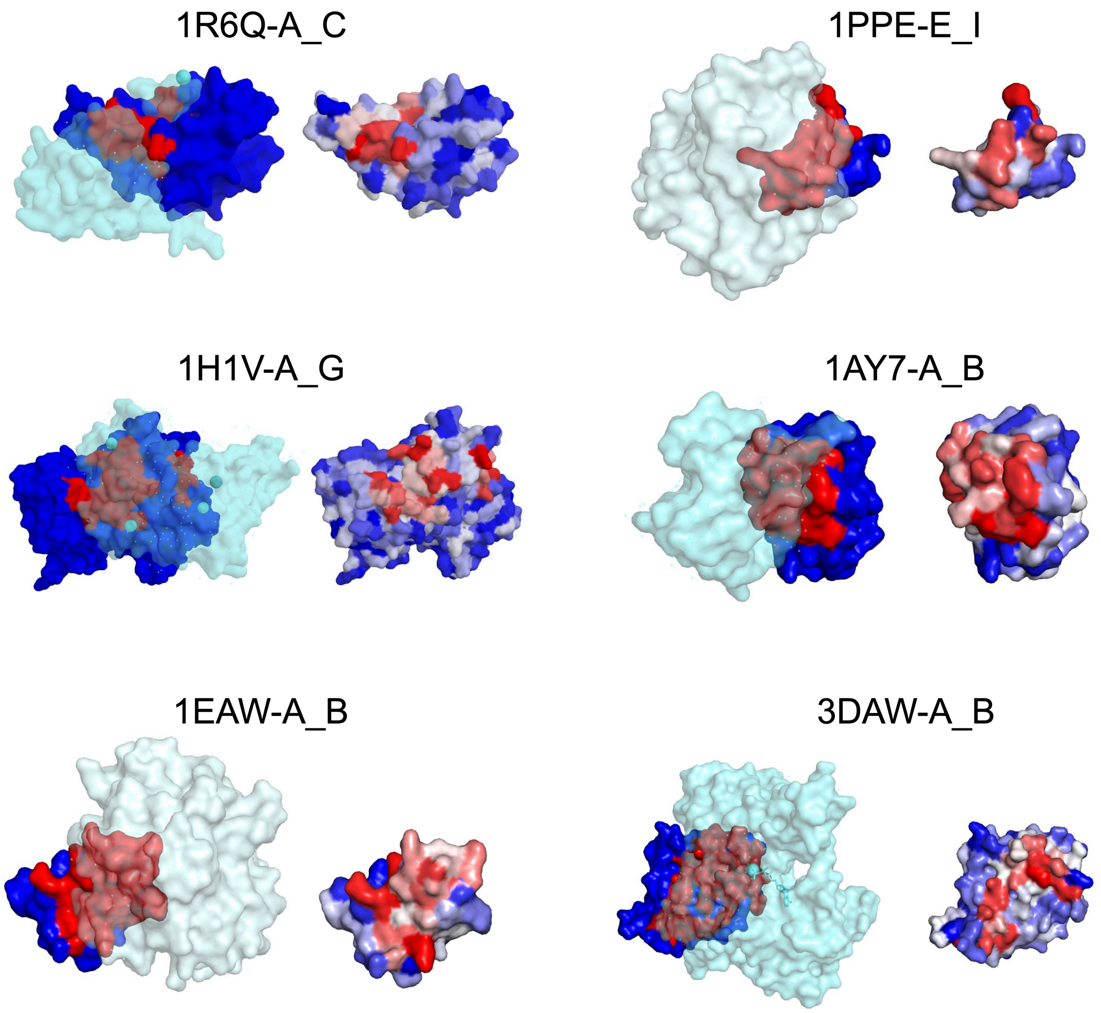

Supplementary Fig. 7. Interpretability of HIGH-PPI for identifying binding surfaces mapped onto the 'surface' models of tested PPI pairs of the 'binding' type. Residues of the query protein learned (right) from HIGH-PPI is colored ranging from low (blue) to high (red) importance. The ground truth (left) of binding surfaces highlighted in red is retrieved from the PDBePISA. Source data are provided as a Source Data file.

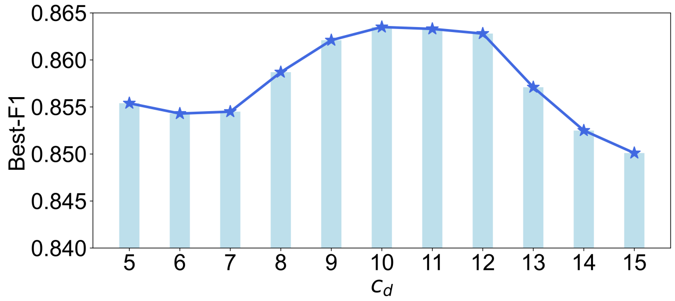

Supplementary Fig. 8. Experimental results for optimal cutoff distance (c_d) selection. Source data are provided as a Source Data file.

Dataset	Data 1	Data 2	Data 3	Data 4	Data 5	Data 6	Data 7	Data 8	Data 9
FPR_{train} / FNR_{train}	**0.1/0.1**	**0.2/0.2**	**0.3/0.3**	**0.4/0.4**	**0.5/0.5**	**0.6/0.6**	**0.7/0.7**	**0.8/0.8**	**0.9/0.9**

Supplementary Table 1. Statistics of FPR_{train} and FNR_{train} of 9 datasets created for training.

Seeds		1	2	3	4	5	6	7	8	p_{value}	pt
**FPR**	**Bl**	0.499	0.490	0.529	0.549	0.546	0.516	0.532	0.527	**0.03842**	NS
	**Ours**	0.530	0.517	0.501	0.482	0.487	0.506	0.495	0.494		
**FNR**	**Bl**	0.200	0.187	0.181	0.177	0.191	0.181	0.181	0.194	**0.00012**	S
	**Ours**	0.132	0.130	0.138	0.142	0.115	0.119	0.140	0.153		
**FDR**	**Bl**	0.199	0.181	0.200	0.207	0.221	0.190	0.201	0.211	**0.00015**	S
	**Ours**	0.147	0.138	0.138	0.134	0.110	0.122	0.138	0.150		

Supplementary Table 2. Experimental results on reliable datasets. We show the results of three metrics after running 8 seeds and obtain the p_{value} and permutation test decision pt, where 'S' represents significant performance and 'NS' represents the performance is not significant. 'Bl' and 'Ours' represent the solid baseline method and ours, respectively. A highlighted 'Ours' represents HIGH-PPI model achieves better performance in terms of the average score across 8 seeds. We perform two-sided t-test for significance analysis.

Unreliable data		Data 1	Data 2	Data 3	Data 4	Data 5	Data 6	Data 7	Data 8	Data 9	Δ	p_{value}	pt
**FPR**	**Bl**	0.537	0.570	0.581	0.604	0.620	0.602	0.657	0.739	0.717	0.180	**0.00004**	S
	**Ours**	0.505	0.526	0.545	0.555	0.563	0.575	0.621	0.655	0.665	0.160		
**FNR**	**Bl**	0.192	0.266	0.306	0.348	0.368	0.422	0.440	0.565	0.698	0.506	**0.00410**	S
	**Ours**	0.171	0.180	0.199	0.213	0.236	0.262	0.362	0.599	0.641	0.470		
**FDR**	**Bl**	0.216	0.324	0.380	0.449	0.487	0.524	0.600	0.787	0.854	0.638	**0.00032**	S
	**Ours**	0.174	0.196	0.229	0.252	0.285	0.324	0.482	0.739	0.780	0.606		

Supplementary Table 3. Experimental results on unreliable datasets. We show the results of three metrics after testing the model on reliable test set labels and obtain the p_{value} and permutation test decision pt, where 'S' represents significant performance and 'NS' represents the performance is not significant. Besides, 'Bl' and 'Ours' represent the methods GNN-PPI and HIGH-PPI, respectively. A highlighted 'Ours' represents the HIGH-PPI model achieves better performance in terms of the average score across 9 unreliable datasets. Δ represents the difference between the maximum and minimum value. We perform two-sided t-test for significance analysis.

Supplementary Method 1: The process for selecting 7 important features out of 12 options.

We outline the process to choose 7 crucial features out of 12 available options, and we provide explanations based on domain knowledge in physicochemical processes. All accessible properties (with links) that we can find at the amino acid level for preparation are listed in Supplementary Table 6.

No.	Access	Feature name	Selected?
1	IPC	Isoelectric Point	√
2	Wikipedia	Polarity	√
3	Wikipedia and MolGpKa	Acidity and Alkalinity	√
4	RDKit	Hydrogen Bond Acceptor	√
5	RDKit	Hydrogen Bond Donor	√
6	RDKit	Octanol-Water Partition Coefficient	√
7	RDKit	Topological Polar Surface Area	√
8	Wikipedia	Relative Abundance	×
9	Wikipedia	Relative Molecular Mass	×
10	Website	Van Der Waals Volume	×
11	Wikipedia	Number of Rotatable Keys	×
12	RDKit	Number of Aromatic Rings	×

Supplementary Table 4. Easily available features of residue level as optional inputs for our model.

Feature selection experiments:

Here, we humbly offer a succinct explanation of feature selection in this work. AI models can become more accurate and run faster by selecting the best subset of input information. Extremely fast feature selection is made possible by well-known model-independent methods such as maximizing correlation coefficient and maximizing mutual information. The model-independent feature selection methods, however, can only estimate the performance of AI models up to a certain point and might not be appropriate for all of them. Despite the potential time commitment, we employ model training-based methodologies to choose the best subset of features for our proposed model. Below, we describe the model-dependent feature selection approach.

Experiment process: To train and test our model, we remove a specific feature dimension from the dataset (note: not zero padding). We run 3 seeds for each feature dimension and determine the feature importance based on the average best-F1 score's negative value. All 12 optional features' importance values are obtained, and their z-scores are then computed.

Results: In Supplementary Table 7, we display the mean of the importance for each kind of feature. We also show the z-scores and the final sort results. Following the sort result, we gradually increase the feature dimension from Topological Polar Surface Area (ranked 1st). The AUPR and F1 results peak once the feature of Octanol-Water Partition Coefficient (ranked 7th) is included. Thus, we ultimately settled on the seven physicochemical properties shown in the manuscript.

**Removed feature**	**Best-F1 ↓**	**z-score ↑**	**Sort result**	**Selected?**
Topological Polar Surface Area	84.25	1.56	1	√
Isoelectric Point	84.44	1.25	2	√
Hydrogen Bond Donor	84.57	1.04	3	√
Polarity	84.83	0.62	4	√
Hydrogen Bond Acceptor	85.00	0.35	5	√
Acidity and Alkalinity	85.12	0.16	6	√
Octanol-Water Partition Coefficient	85.40	-0.30	7	√
Van Der Waals Volume	85.52	-0.49	8	×
Number of Aromatic Rings	85.57	-0.57	9	×
Number of Rotatable Keys	85.69	-0.76	10	×
Relative Abundance	86.08	-1.40	11	×
Relative Molecular Mass	86.13	-1.48	12	×

Supplementary Table 5. Average F1 scores of our model after dropping each feature. We then calculate the importance z-scores for ranking. ↓ means lower Best-F1 score corresponds to more important feature. Instead, ↑ means higher z-score corresponds to more important feature.

Supplementary Method 2: A detailed discussion for introducing protein domain information into the hierarchical model.

Protein domain may offer a useful middle-scale view on the PPI issue. From both structural and functional standpoints, protein domains fall somewhere in the middle of amino acids and proteins. Protein domains are compact, foldable, three-dimensional structures made of amino acid residues. The three-dimensional structure of the complete protein is made up of several protein domains acting as structural building blocks. When it comes to function, each domain oversees expressing a certain protein function. The specificity of protein activities is produced by the assembly of various domains, which also controls the existence or absence of PPIs and hot spots at the PPI interface. Therefore, both in terms of structures and functions, the protein domain represents a crucial middle scale for the PPI problem.

Although protein domain information is important, to our knowledge, true (native) domain annotations are not easily available and usually come from computational softwares, which inevitably leads to the data unreliability. Nevertheless, we respectfully recommend further research on multi-view hierarchical learning for PPI prediction.

About future work: ① (Information benefits) It has been demonstrated that the proposed double-viewed hierarchical model, which is based on the high degree of information complementarity between the data of two views, benefits from both views. For instance, the PPI network (top view) will suggest active protein information (*i.e.,* with a high network degree from the top view) to the bottom view. Similarly, the bottom view helps PPI network to complement residue-level fragmentation knowledge, *i.e.,* the knowledge of the functions performed by residue fragments for a particular PPI instance. The two current perspectives will benefit from the accuracy of the domain annotations because it provides data on functional residue segments that are crucial to PPIs. However, when the domain scale is employed as a separate view, data unreliability could spread to other views and impair the hierarchical model as a whole. Therefore, for the information gain in the hierarchical model, an early reliability assessment of the available domain annotations is required. ② (Supervised information for bottom residue-scale view) HIGH-PPI now enables interpretable identification for PPI-related functional sites without supervising domain data. Therefore, directly supervising the selection of significant functional sites is a straightforward way for the double-viewed hierarchical model to gain from domain information. Precisely, a well-designed regularization is required to guarantee that all functional sites, discovered by HIGH-PPI, belong in the prepared domain database. The domain regularization and the PPI prediction loss form a flexible trade-off of learning objectives, which can appropriately tolerate the domain annotation unreliability. ③ (Computational efficiency) It is worth mentioning that the explosion of data required for multi-view hierarchical modeling necessitates the use of lightweight backbones for quick training and prediction. Our suggested hierarchical graph learning backbone, a memory and compute efficient backbone for simultaneously learning the structure-function relationship, may therefore provide insight for future studies. ④ (Protein structure modeling) The research on protein structure modeling, which is critical for understandable mining of essential interaction areas, needs to be continued in the upcoming work.

Supplementary Method 3: A detailed experimental protocol for evaluating the model performance on test pairs of three distinct class.

Experimental protocol:

There're following tips to clarified before the experiments:

First, we created 3 data partitions based on the SHS27k dataset. Among the test sets split by 3 data partitions, each of them shares a range of 3 different test pairs including C_1, C_2, and C_3. Here, C_1 stands for the percentage of PPIs of which **both proteins were present** in a training set (Class 1), C_2 stands for the percentage of PPIs of which **either of (but not both) proteins was present** in the training set (Class 2), C_3 stands for the percentage of PPIs of which **neither protein was present** in the training set (Class 3).

Second, we didn't directly employ the random or OOD (BFS, DFS) partitioning methods presented in the manuscript to create the three partitions because of two limitations: 1) the C_3 from random partitioning is almost 0%, and 2) the C_1 from OOD partition is almost 0%, thus neither of which are suitable for statistical evaluation of **3 distinct classes**. To overcome this issue, we combine the random and OOD partitioning methods to ensure that none of C_1, C_2, and C_3 is considerably smaller than the other two.

Third, the 3 datasets were directly used in the experiments of generalization evaluation (GE) to assess the model's capacity to handle OOD data. Moreover, we additionally added perturbations to the training sets of the created 3 OOD datasets and performed robustness evaluation (RE).

Generalization evaluation experiments: We train and test the model on the 3 produced datasets. For each dataset, we show model performance on the overall dataset and on 3 distinct classes. For each experiment, we run 5 seeds and report the average F1 score.

Robustness evaluation experiments: We follow the experimental protocol for robustness evaluation in the manuscript. As for the 3 produced OOD datasets, we add random perturbations (perturbation ratio is 0.2) on their training sets and retain their test sets unmodified. For each dataset, the model's performance is displayed in overall fashion and respective fashion on 3 distinct classes. For each experiment, we run 5 seeds, and then report the average F1 score.

Results and discussions:

**Dataset**	$C_1/C_2/C_3$ **(%)**	**Overall**		**Class 1**		**Class 2**		**Class 3**	
		Ours	Bl	Ours	Bl	Ours	Bl	Ours	Bl
**1**	27/51/22	75.05	70.77	87.22	84.12	78.31	73.20	52.56	48.77
**2**	36/48/16	77.40	72.17	87.46	84.53	77.40	71.19	54.79	47.28
**3**	21/64/15	75.94	71.48	87.29	84.43	77.09	72.94	55.17	47.09
**Avg. gain**		4.40		2.96		5.16		6.46	

Supplementary Table 6. Generalization experiments on 3 distinct classes. We show the averaged

F1 scores of our model and a strong baseline across 5 seeds. ‘Bl’ and ‘Ours’ represent the solid baseline method (GNN-PPI) and HIGH-PPI, respectively. Avg. gain shows the performance gain of our method on the overall and on 3 classes.

**Dataset**	$C_1/C_2/C_3$ **(%)**	**Overall**		**Class 1**		**Class 2**		**Class 3**	
		Ours	Bl	Ours	Bl	Ours	Bl	Ours	Bl
**1**	27/51/22	63.42	56.21	81.13	73.91	62.75	55.89	43.26	35.26
**2**	36/48/16	66.23	59.72	80.52	73.63	61.17	56.59	45.40	37.79
**3**	21/64/15	63.75	56.57	80.44	73.05	62.53	55.70	45.62	37.24
**Avg. gain**		6.97		7.16		6.09		8.00	

Supplementary Table 7. Robustness experiments on 3 distinct classes. We show the averaged F1 scores of our model and a strong baseline across seeds. ‘Bl’ and ‘Ours’ represent the solid baseline method (GNN-PPI) and HIGH-PPI, respectively. Avg. gain shows the performance gain of our method on the overall and on 3 classes.

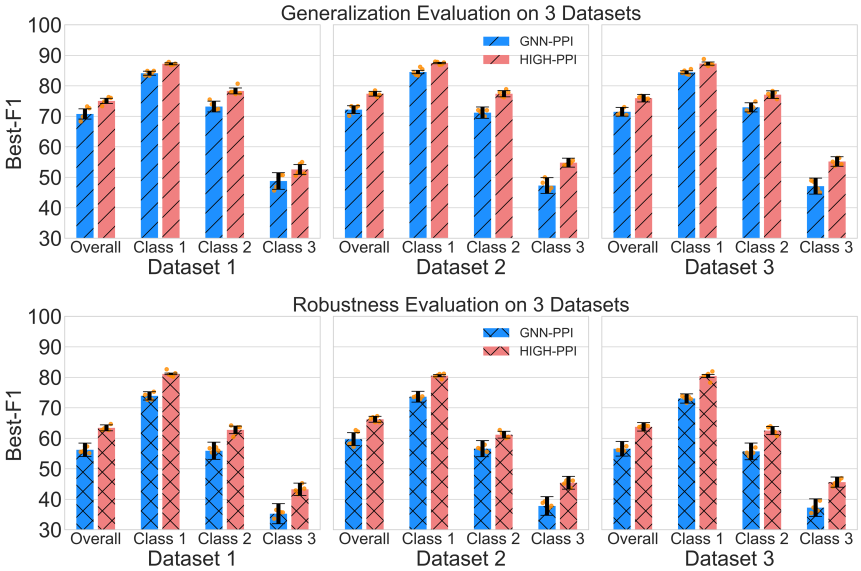

Supplementary Fig. 9. Bar chart visualization for Supplementary Table 5 and 6. Error bars represent standard deviation of the mean (under 5 independent runs). Source data are provided in Supplementary Table 6 and 7.

Additional findings:

(1) In GE, our method performs better on all occasions we presented. As can be seen on the Avg. gain, as the prediction difficulty increases (Class: 1→2→3), the superiority of our method tends to be more significant (Avg. gain: 2.96→5.16→6.46), demonstrating that our model could better handle OOD protein pair data.

(2) RE is essentially a robust test of the model using OOD data, in which hence the models performed even significantly worse than they do in GE. Even for the easy-to-predict (Class 1) test pairs, RE leads to a 10.83 drop in the average performance score compared to GE.

(3) Compared to GE, our model has a more significant advantage than in RE. The advantage is reflected in the overall (Avg. gain: 4.40 vs. 6.97) and 3 distinct classes (Avg. gain: (2.96, 5.16, 6.46) vs (7.16, 6.09, 8.00)). This suggests that our model can better handle the most realistic scenarios of PPI problem, where both OOD and perturbations are present.
