# Supplementary material for: Hierarchical graph learning for protein–protein interaction
Source: Nat Commun. 2023 Feb 25;14:1093. doi: 10.1038/s41467-023-36736-1 (PMC9968329; doi:10.1038/s41467-023-36736-1)
Supplement: Supplementary file 3 — Description of Supplementary Data [file 41467_2023_36736_MOESM3_ESM.docx]

**Description of Supplementary Data Files**

**Supplementary Data 1:** ENSP numbers and amino acid sequences for all selected proteins of the SHS27k dataset.

**Supplementary Data 2:** Ground truth results and HIGH-PPI predictions for all test PPIs of the SHS27k dataset.

**Supplementary Data 3:** Quantitative values of seven types of properties for each amino acid.
