# Supplementary material for: Hierarchical graph learning for protein–protein interaction
Source: Nat Commun. 2023 Feb 25;14:1093. doi: 10.1038/s41467-023-36736-1 (PMC9968329; doi:10.1038/s41467-023-36736-1)
Supplement: Supplementary file 7 — Reporting Summary [file 41467_2023_36736_MOESM7_ESM.pdf]

nature portfolio

Corresponding author(s): Yong Huang and Jia Li

Last updated by author(s): Jan 27, 2023

Reporting Summary

Nature Portfolio wishes to improve the reproducibility of the work that we publish. This form provides structure for consistency and transparency in reporting. For further information on Nature Portfolio policies, see our Editorial Policies and the Editorial Policy Checklist.

Statistics

For all statistical analyses, confirm that the following items are present in the figure legend, table legend, main text, or Methods section.

n/a	Confirmed	
☐	☒	The exact sample size (*n*) for each experimental group/condition, given as a discrete number and unit of measurement
☐	☒	A statement on whether measurements were taken from distinct samples or whether the same sample was measured repeatedly
☐	☒	The statistical test(s) used AND whether they are one- or two-sided<br>*Only common tests should be described solely by name; describe more complex techniques in the Methods section.*
☒	☐	A description of all covariates tested
☐	☒	A description of any assumptions or corrections, such as tests of normality and adjustment for multiple comparisons
☐	☒	A full description of the statistical parameters including central tendency (e.g. means) or other basic estimates (e.g. regression coefficient) AND variation (e.g. standard deviation) or associated estimates of uncertainty (e.g. confidence intervals)
☐	☒	For null hypothesis testing, the test statistic (e.g. *F*, *t*, *r*) with confidence intervals, effect sizes, degrees of freedom and *P* value noted<br>*Give P values as exact values whenever suitable.*
☒	☐	For Bayesian analysis, information on the choice of priors and Markov chain Monte Carlo settings
☐	☒	For hierarchical and complex designs, identification of the appropriate level for tests and full reporting of outcomes
☐	☒	Estimates of effect sizes (e.g. Cohen's *d*, Pearson's *r*), indicating how they were calculated

Our web collection on statistics for biologists contains articles on many of the points above.

Software and code

Policy information about availability of computer code

Data collection	We used the following tools for data collection:<br>PDBePISA v1.48<br>Protein Data Bank v50<br>AlphaFold v2.3.1<br>Catalytic Site Atlas v2.0<br>STRING v11.5<br>UniProt v2022_05
Data analysis	HIGH-PPI framework was implemented by the Pytorch v1.11.0 library. The operating system version is Ubuntu v16.04. The python version is v3.7.0. Analysis was performed by numpy v1.21.5, scipy v1.6.2 and pandas v1.4.1. More details can be found in the 'Methods' section, supplementary information and the code repository https://github.com/zqgao22/HIGH-PPI. We computed residue importance with GNNExplainer (the Deep Graph Library version) https://github.com/ShaharGottlieb/GNNExplainer_DGL.

For manuscripts utilizing custom algorithms or software that are central to the research but not yet described in published literature, software must be made available to editors and reviewers. We strongly encourage code deposition in a community repository (e.g. GitHub). See the Nature Portfolio guidelines for submitting code & software for further information.

Data

Policy information about availability of data

All manuscripts must include a data availability statement. This statement should provide the following information, where applicable:

- Accession codes, unique identifiers, or web links for publicly available datasets
- A description of any restrictions on data availability
- For clinical datasets or third party data, please ensure that the statement adheres to our policy

The PPI and protein data used in this study are available in the Zenodo database under accession code https://doi.org/10.5281/zenodo.7213401. They are obtained from the following publicly available database. Datasets containing protein sequences and their interaction annotations are obtained from https://github.com/muhaochen/seq_ppi. The native protein structures are obtained from PDB: https://www.rcsb.org/. Protein structures with errors are obtained from AlphaFold: https://alphafold.ebi.ac.uk/. The catalytic site information of proteins can be found at CSA: https://www.ebi.ac.uk/thornton-srv/m-csa/. The ground truth of binding site information is obtained from PDBePISA: https://www.ebi.ac.uk/pdbe/pisa/. Source data are provided with this paper.

Human research participants

Policy information about studies involving human research participants and Sex and Gender in Research.

Reporting on sex and gender	This is not relevant to our study.
Population characteristics	This is not relevant to our study.
Recruitment	This is not relevant to our study.
Ethics oversight	This is not relevant to our study.

Note that full information on the approval of the study protocol must also be provided in the manuscript.

Field-specific reporting

Please select the one below that is the best fit for your research. If you are not sure, read the appropriate sections before making your selection.

[x] Life sciences [ ] Behavioural & social sciences [ ] Ecological, evolutionary & environmental sciences

For a reference copy of the document with all sections, see nature.com/documents/nr-reporting-summary-flat.pdf

Life sciences study design

All studies must disclose on these points even when the disclosure is negative.

Sample size	All the datasets for training and evaluating our model are available from existing databases. We use 3 datasets called SHS27k, SHS148k and STRING. They contain 7624, 44488, and 593397 PPI samples, respectively. Chosen datasets are sufficient for the analysis because: (1) the sizes are chosen to ensure that the native structures of each protein in the training set is available; (2) memory and computation efficiency are important determinants; (3)for each PPI type, no less than 100 samples are used in the dataset.
Data exclusions	For training our model to the best level, very few proteins whose native structures are unavailable were excluded from the training set.
Replication	Experiments were replicated every five days for approximately two months prior to submission. All attempts at replication were successful.
Randomization	This is not relevant to our study because we did not make quantitative comparisons between groups of samples.
Blinding	This is not relevant to our study because there was no group allocation nor sample comparison.

Reporting for specific materials, systems and methods

We require information from authors about some types of materials, experimental systems and methods used in many studies. Here, indicate whether each material, system or method listed is relevant to your study. If you are not sure if a list item applies to your research, read the appropriate section before selecting a response.

Materials & experimental systems

n/a	Involved in the study
☒	☐ Antibodies
☒	☐ Eukaryotic cell lines
☒	☐ Palaeontology and archaeology
☒	☐ Animals and other organisms
☒	☐ Clinical data
☒	☐ Dual use research of concern

Methods

n/a	Involved in the study
☒	☐ ChIP-seq
☒	☐ Flow cytometry
☒	☐ MRI-based neuroimaging
